# Revisiting Community Case Management of Childhood Pneumonia: Perceptions of Caregivers and Grass Root Health Providers in Uttar Pradesh and Bihar, Northern India

**DOI:** 10.1371/journal.pone.0123135

**Published:** 2015-04-21

**Authors:** Shally Awasthi, Mark Nichter, Tuhina Verma, Neeraj Mohan Srivastava, Monica Agarwal, Jai Vir Singh, CAP-Lucknow Team

**Affiliations:** 1 Department of Pediatrics, King George’s Medical University, Lucknow, India; 2 The School of Anthropology, University of Arizona, Tucson, United States of America; 3 UNICEF’s Office for Uttar Pradesh, Lucknow, India; 4 Department of Community Medicine, King George’s Medical University, Lucknow, India; Centre Hospitalier Universitaire Vaudois, FRANCE

## Abstract

**Background:**

Community-acquired pneumonia (CAP) is the leading cause of under-five mortality globally with almost one-quarter of deaths occurring in India.

**Objectives:**

To identify predisposing, enabling and service-related factors influencing treatment delay for CAP in rural communities of two states in India. Factors investigated included recognition of danger signs of CAP, health care decision making, self-medication, treatment and referral by local practitioners, and perceptions about quality of care.

**Methods:**

Qualitative research employing case studies (CS) of care-seeking, key informant interviews (KII), semi-structured interviews (SSI) and focus group discussions (FGD) with both video presentations of CAP signs, and case scenarios. Interviews and FGDs were conducted with parents of under-five children who had suffered CAP, community health workers (CHW), and rural medical practitioners (RMP).

**Results:**

From September 2013 to January 2014, 30 CS, 43 KIIs, 42 SSIs, and 42 FGDs were conducted. Recognition of danger signs of CAP among caregivers was poor. Fast breathing, an early sign of CAP, was not commonly recognized. Chest in-drawing was recognized as a sign of serious illness, but not commonly monitored by removing a child’s clothing. Most cases of mild to moderate CAP were brought to RMP, and more severe cases taken to private clinics in towns. Mothers consulted local RMP directly, but decisions to visit doctors outside the village required consultation with husband or mother-in-law. By the time most cases reached a public tertiary-care hospital, children had been ill for a week and treated by 2-3 providers. Quality of care at government facilities was deemed poor by caregivers.

**Conclusion:**

To reduce CAP-associated mortality, recognition of its danger signs and the consequences of treatment delay needed to be better recognized by caregivers, and confidence in government facilities increased. The involvement of RMP in community based CAP programs needs to be investigated further given their widespread popularity.

## Introduction

Pneumonia is the single largest cause of mortality in children under-five years of age, responsible for one in five deaths [1]. In 2010, there were an estimated 1 3 million deaths due to childhood pneumonia, one-quarter being in India. Among these, 81% of the deaths occurred in the first two years of life [2].

Poor and delayed care-seeking has been implicated in 6–70% of child deaths in developing countries, including those from pneumonia [3,4]. Available literature attributes poor care-seeking, including delay or no care-seeking, to various social, cultural and logistical barriers at the household, community and health system levels which often interact. [5,6]. One important factor possibly delaying qualified care seeking is the community’s trust in treatment provided by the easily accessible but largely unqualified rural medical practitioners (RMP) [7,8].

Childhood pneumonia, if recognised early, is easily treatable through low-cost antibiotics. However, once it gets severe, the out-of-pocket health expenditures often turn catastrophic [9]. In 2010, globally there were an estimated 120 million episodes of childhood pneumonia, 14 million of which progressed to be severe [2]. Thus, prompt and appropriate care-seeking for childhood pneumonia has implications both in terms of child survival as well as the expenditures at the household and the health system levels.

There is sparse evidence on community-based management (including care-seeking) of childhood pneumonia in India [10]. Available evidence suggests that recognition of pneumonia and its danger signs indicating disease worsening was low, home/traditional remedies were often used for specific signs of pneumonia (such as, chest in-drawing and fast breathing) and the RMPs were the primary health providers [11–13].

To achieve the Millennium Development Goal (MDG) 4 in low-income countries increased investment in primary health care as well as health system strengthening is required. Community involvement and outreach requires behaviour change as well as active participation of community health workers (CHWs) to provide effective primary health care to achieve MDG 4 [14]. In order to develop effective outreach programs for community-acquired pneumonia (CAP) it is imperative to learn more about community recognition of pneumonia and factors contributing to health care seeking delay as well as to assess the potential role of CHWs in future education programs [15].

## Materials and Methods

### Ethics Statement

This study was approved by the Institutional Ethics Committee, King George’s Medical University (KGMU), Lucknow and relevant public authorities. Written, informed consent was taken from the study participants prior to the conduct of interviews, discussions and making videos. Participants were briefed about the research purpose, methods and involvement at the time of obtaining consent.

### Study Setting

This study was carried out in Uttar Pradesh (UP) and Bihar, the two states of northern India that have high under-five mortality rates, per 1000 live births, of 90 and 70 respectively, as compared to the national average of 52 [16,17]. Five districts (Meerut, Agra, Lucknow, Mahoba and Gorakhpur) in UP and two districts (Darbhanga and Gaya) in Bihar were purposively selected based on their local dialects. Dialects spoken in these districts are: *Awadhi* (Lucknow), *Bhojpuri* (Gorakhpur), *Bundelkhandi* (Mahoba), *Braj* (Agra), *Khari Boli* (Meerut), *Maghai* (Gaya) and *Maithali* (Darbhanga). There was a high prevalence of any acute respiratory infection among under-five children (within two weeks preceding the survey) in the project districts, ranging from 11.6% in Gorakhpur to 29.7% in Darbhanga [16].

**Fig 1:**
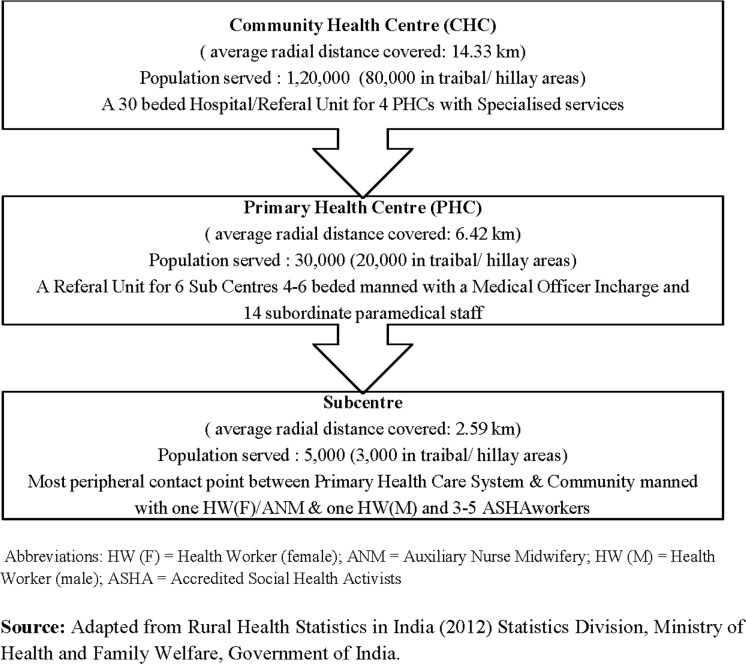
Rural Health Care System in India.

A three-tier primary health care system comprising with Subcentre, Primary Health Centre (PHC) and Community Health Centre (CHC) exists in rural UP and Bihar (Fig 1). Apart from these health care units, there are Accredited Social Health Activists (ASHAs) at the village, who are community-based workers providing preventive health services to a population of approximately 1000. In the private sector, mostly unqualified RMPs are present at almost every village. Private qualified doctors (clinics/hospitals) are usually found only at the block and district levels, along with the public health system facilities and providers.

### Study Design

**Fig 2:**
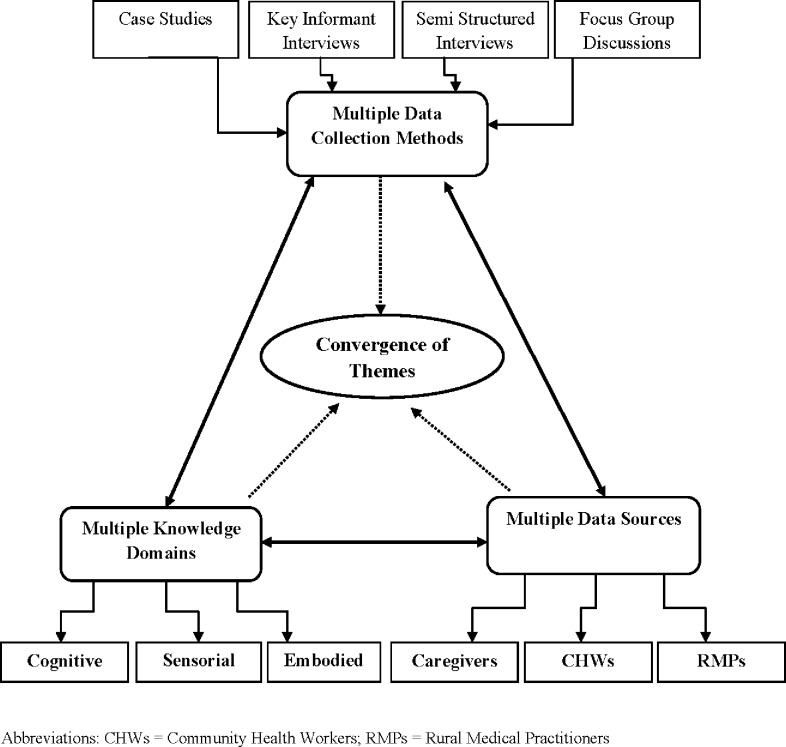
Triangulation Plan.

The methods selected for use in this study were adapted from the Focused Ethnographic Study (FES) methodology recommended for community-based Acute Respiratory Infections (ARI) research by the World Health Organization, which have been field tested in several different countries including India [18–20].The current study employed Case Studies (CS), Key Informant Interviews (KIIs), Semi-structured Interviews (SSIs), Focus Group Discussions (FGDs) and projective case scenarios. Use of multiple qualitative methods for obtaining information from multiple sources and evoking recall by using audio-visual as well as auditory stimuli (in for form of scenario narrations) ensured triangulation of information obtained and its internal validity [21]. Triangulation of methods designed to capture cognitive, embodied, sensorial and experience based knowledge has been demonstrated to be particularly important when studying CAP related symptom recognition and perceptions of illness severity [20]. The triangulation plan of the study is presented in Fig 2.

Case studies documented the deliberations and actions of the parents of sick children admitted for CAP in selected tertiary care facilities. Interviews focused on CAP symptom and severity recognition, care seeking, care provision [22], and determinants of health care decision making and delay [23]. Hypothetical case scenarios drew on themes found in actual cases and were employed in interviews and focus groups to probe what respondents deemed reasonable courses of action when presented with different hypothetical cases of childhood CAP. Interviews focused on the local language used to describe signs and symptoms of CAP, the past experience of child caretakers when confronted with illness symptoms they recognised as dangerous, the social relations and economic realities of health care decision making, and health care workers’ and local practitioners’ treatment of respiratory illnesses and patterns of referral. Next, videos of children having different levels of severity of CAP were shown to informants as a means to move beyond language to recognition and sensorial assessment of visual and auditory signs. KIIs, SSIs, and FGDs were conducted with the caregivers of children (mothers, grandmothers, female relatives and fathers), KIIs and FGDs with community health workers (ASHAs, ANMs) and KIIs with RMPs.

Researchers were trained in the methods employed in the study by a consultant having experience applying the FES in South India [19] and health social scientists he had formerly trained during an INCLEN/WHO Zinc and diarrheal disease study [24]. Fieldworkers employed for this study attended a “hands on” training workshop which included clinic based interviewing exercises. Their research was carefully monitored in the field by senior social scientist who accompanied them to the field for the first few weeks of the project, and the consultant who made field visits during different stages of research. Ongoing monitoring and assessment of field notes took place and inter-rater reliability of data coding was ensured by different team members, including the PI, cross checking how data was coded. Triangulation of data captured by different methods was enabled by a common set of codes developed after a representative selection of interviews were analyzed and code definitions established.

### Data Collection

The study was conducted from September 2013 till January 2014 in four tertiary care hospitals and four villages in each of 7 districts in UP and Bihar.

#### Case Studies

A set of thirty pneumonia case studies where there was a bad outcome, like prolongation of hospital stay or development of complications or death were compiled. Caregivers (mother/ father/close relative) of such a case, aged one month to five years were interviewed after obtaining written, informed consent. A pre-designed and pretested interview guide was used for data collection. Recruitment continued till saturation was obtained.

#### Selection of Blocks, Sub-centres and Villages

List of all the blocks within each district was obtained. One block per district was purposively selected. The blocks selected in project districts were: *Manpur* block in Gaya, *Bahadurpur* block in Darbhanga, *Bakshi Ka Talab* block in Lucknow, *Sahjanwan* block in Gorakhpur, *Fatehpur Sikri* block in Agra, *Charkhari* block in Mahoba and *Mawana* block in Meerut. Selected blocks were at a distance of 20–25 km from the respective district head quarters.

After this, the study team visited the CHCs in each of these blocks in UP. However, since there were no CHCs in Bihar and only a Main PHC along with its additional PHCs at the block level, the team visited the main PHCs in the districts of Bihar. From the list of sub-centres functional within the selected CHCs/PHCs, four to six sub-centres in four corners of the CHCs were identified and selected. Thereafter, 1–2 villages were randomly selected within each sub-centre and visited by the team for data collection.

#### Identification and selection of respondents

Caregivers: For the identification and selection of caregivers for KIIs, SSIs and FGDs, the team visited the sampled villages. Assistance of ASHAs of the respective villages was sought to identify the respondents. At places where ASHAs were unavailable, the village head, *“Pradhan”* or any other influential person from the community was approached for identifying respondents.

CHWs: The official list of sub-centres was obtained which included names of ANMs and ASHAs posted. KIIs and FGDs were conducted with the CHWs at their respective CHC/PHC. One FGD per district, with 6–8 CHWs, was conducted at the CHC/PHC of the selected block. CHWs that were already included for KIIs were not included in FGDs.

RMPs: Caregivers were asked to identify the RMP most frequently visited by them. This RMP was then interviewed at their respective clinic.

#### Eligibility Criteria

Included were local residents, giving consent in writing for participation. Those caregivers were included who had a child <5 years of age in the family, healthy on the day of interview, but had witnessed one episode of acute respiratory illness in a child <5 years of age in past 6 months for which medical treatment was availed. Specific eligibility criterions of the respondents are presented in Table 1.

**Table 1:** Eligibility Criteria of the Respondents.

Data Collection Technique	Respondents Category	Specific Eligibility Criteria
KIIs	Younger Caregiver	Married woman aged 30–45 years; Mother of at least ONE child aged 1 month to 5 years
KIIs	Older Caregiver	Married woman aged 45–60 years; Grandmother or any other female relative of a family in which there is a child between 1 month to 5 years
SSIs	Younger Caregiver	Married woman ≤30 years; Mother of at least ONE child aged 1 month to 5 years
SSIs	Older Caregiver	Married woman >30 years; Mother or grandmother or any other female relative of a family in which there is a child between 1 month to 5 years
FGDs	Younger Caregiver	Married women ≤30 years
FGDs	Older Caregiver	Married women >30 years
FGDs	Father	Married men between 28–45 years
KIIs	Community Health Worker	Auxiliary Nurse Midwife (ANM) or a Accredited Social Health Activists (ASHA) in service for 2 years or more
KIIs	Rural Medical Practitioner	Person practicing in the area for atleast 2 years & whose exact qualifications are not known
FGDs	Community Health Worker	Auxiliary Nurse Midwife (ANMs) or Accredited Social Health Activists (ASHAs) in service for 2 years or more

Abbreviations: KIIs = Key Informant Interviews; SSIs = Semi-structured interviews; FGDs = Focused Group Discussions

#### Research Instruments

Interview guides were prepared for conducting KIIs, SSIs and FGDs. Each interview schedule was first developed in English and then translated into Hindi. These schedules were pilot tested in the hospital as well as a rural setting in Lucknow with each of the respondent categories.

Illness scenarios and clinical illness videos, photographed after written informed consent of the parent, were used during the discussion/interview to elicit responses on actions the respondent(s) would take in such situations. Three hypothetical illness scenarios have been described in Table 2 **.** Often scenarios gave rise to broader discussion about health care-seeking choices in a particular locale, seasonality and so on. The FGD respondents were presented a video presentation of signs of CAP also written in Table 2.

**Table 2:** Hypothetical Case Scenarios and Video Presentations of Signs of CAP.

Level of CAP severity	Hypothetical Case Scenarios used in Key Informant Interviews and Semi Structured Interviews	Video Presentations used in Focus Group Discussions
Mild to Moderate	**Illness Scenario I:** Child less than 5 years has cough, runny nose and was warm to touch. He/she is otherwise healthy and is also feeding normally.	**Video I:** A child less than 5 years presenting with only fast breathing (an early sign of pneumonia)
Severe	**Illness Scenario II:** Child less than 5 years has cough, runny nose, fever, fast breathing and chest in-drawing. He/she is feeding / breastfeeding less than usual.	**Video II:** A child less than 5 years presenting with chest in-drawing along with fast breathing and difficult breathing
Very Severe	**Illness Scenario III:** Child less than 5 years has cough, runny nose fever, difficulty in breathing and chest in-drawing. He/she is unable to drink/ breastfeed normally. He/she has altered sensorium and is also having bluish discoloration of the lips.	**Video III:** A child less than 5 years presenting with chest in-drawing along with fast breathing, difficult breathing, grunting/groaning and altered sensorium

Summarily, we used several methods to study recognition of CAP signs matched against danger signs included on the Integrated Management of Neonatal & Childhood Illnesses (IMNCI) check list, among child caregivers and local health care providers, through probes (SSIs), projective case scenarios (KIIs, SSIs) as well as through clinical illness videos (FGDs). Fast breathing is an IMNCI danger sign of pneumonia while the IMNCI signs of severe pneumonia include general danger signs (inability to feed, lethargy, unconscious, convulsions) of severe disease, chest in-drawing and stridor in a calm child [25].

### Data Management

Transcripts were written by facilitators in Hindi. It was ensured that each transcript was finalised only after referring the field notes and audio recordings. After this, the transcripts were translated into English by a hired translator. Transcriptions were reverse translated in Hindi to capture the real essence of research information. Transcripts and their translations were reviewed by social scientists as well as the project coordinator. Thirty percent of transcripts were reviewed by the Project Investigators.

### Data analysis

#### Codebook

A codebook was developed for coding and data interpretation. Codebook was divided into five heads: (i) Code Level I; (ii) Short Code—Level I; (iii) Code Level II; (iv) Short code—Level II; (v) Definitions of Code level I & II. Code Level I was the main code and the Code Level II was the sub-code that was a part of the broader domain of main code. Code definitions along with levels of codes were discussed and standardized. Code definition also included “when to use” instructions. It gave specific instances, usually based on the data, in which the code should be applied. It also had “when not to use this code” portion which gave instances in which the code could be considered but should not be applied (often because another code would be more appropriate) [26].

#### Coding, Analysis & Interpretation

**Fig 3:**
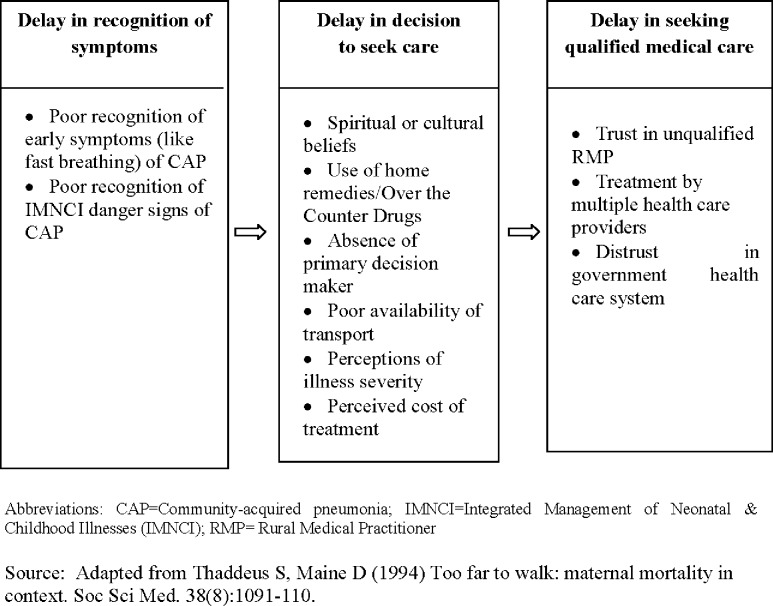
Theoretical Framework of the study.

The theoretical framework of analysis for this study was guided by a model of delay developed by Thaddeus and Maine [27]. The theoretical framework is attached as Fig 3. Manual coding was done to develop concepts and derive key emerging themes. For coding, each transcript was read and re-read by two researchers several times to understand and decide on codes’ allocation. Any discrepancies that occurred during coding was resolved by the analysis team in two ways (a) re-listening to the voice recordings of interviews/discussions and (b) referring to the original transcripts and field notes. The codes were entered in Microsoft Excel for easy management and for counting frequencies. For the purpose of data interpretation, frequency of the responses obtained against each code was reported using a standard term. Concepts emerging from one data collection technique were triangulated with remaining two techniques. Key emerging themes were established based on all emerging concepts. Thematic content analysis approach guided the analysis of the data [28–30]. All data underlying the study findings are available at the project website [31]. The standard terms as used in this paper for frequency of responses obtained against each code are: (a) 100 percent = All; More than 50 percent = Most; 30–50 percent = Almost half; 15–30 percent = some and less than 15 percent = few respondents.

## Results

### Baseline Characteristics

#### Caregivers (n = 303)

From September 2013 to January 2014, we conducted 43 KIIs, 42 SSIs, and 42 FGDs. The baseline characteristics of the study participants are presented in Table 3. We recruited 30 cases, 25 from UP and 5 from Bihar. In two cases the respondents were grandparents, while in all others they were either the mother or the father. All the interviewees had been with the child since the onset of illness. Around half (53.3%) of the interviewees lived in a joint family, majority were from rural areas (76.7%). Majority of the children were infants (76.7%) and males (76.7%).

**Table 3:** Baseline characteristics of caregivers in the KIIs, SSIs and FGDs.

Variables	KIIs	SSIs	FGDs	Total
	(n = 28)	(n = 42)	(n = 233)	(n = 303)
**Religion**				
Hindus	22 (78.6)	38 (90.5)	218 (93.6)	278 (91.8)
Muslims	06 (21.5)	04 (9.4)	14 (6.0)	24 (7.9)
Sikhs	0 (0.0)	0 (0)	1 (0.4)	1 (0.3)
**Caste**				
General	07 (25.0)	14 (33.3)	49 (21.0)	70 (23.1)
Scheduled Castes	06 (21.4)	11 (26.1)	90 (38.6)	107 (35.3)
Scheduled Tribes	01 (3.6)	06 (14.3)	10 (4.3)	17 (5.6)
Other Backward Classes	14 (50.0)	11 (26.2)	84 (36.1)	109 (35.9)
**Age:** Female Caregivers (median (range))	28 (22–36 years)		31 (20–57 years)	
**Age:** Fathers (median (range))*			31 (28–45 years)	
**Family Type**				
Joint	18 (64.3)	26 (61.9)	96 (41.2)	140 (46.2)
Single/nuclear	10(35.7)	16 (38.1)	137 (58.8)	163 (53.8)
**Family Size**				
0–5	4(14.3)	6(14.3)	82 (35.2)	92(30.4)
6–10	16(57.1)	25(59.5)	129 (55.4)	170(56.1)
11–15	7(25.0)	7(16.7)	17 (7.3)	31(10.2)
16 and above	1(3.6)	4(9.5)	5 (2.2)	10 (3.3)
**Education**				
Illiterate	15(53.6)	17(40.5)	63(27.0)	95(31.4)
Literate	1(3.6)	4(9.5)	29(12.5)	34(11.2)
Primary Pass (5 years of education)	2(7.1)	5(11.9)	33(14.2)	40(13.2)
Middle Pass (8 years of education)	6(21.4)	6(14.3)	44(18.9)	56(18.5)
High School (10 years of education)	1(3.6)	4(9.5)	28(12.0)	33(10.9)
Intermediate (12 years of education)	2(7.1)	4(9.5)	21(9.0)	27(8.9)
Graduate	1(3.6)	1(2.4)	11(4.7)	13(4.3)
Post Graduate	0(0.0)	1(2.4)	4(1.7)	5(1.7)
**Occupation**				
Housewife	25 (89.3)	39(92.9)	156 (66.9)	220(72.6)
Farmer	1(3.6)	0 (0.0)	22(9.4)	23(7.6)
Self employed	1 (3.6)	0 (0.0)	8(3.4)	9(3.0)
Service	0 (0.0)	0 (0.0)	9(3.9)	9(3.0)
Labourer	1 (3.6)	2 (4.8)	32(13.7)	35(11.6)
Unemployed	0 (0.0)	0 (0.0)	6(2.6)	6(2.0)
Other	0 (0.0)	1 (2.4)	0(0.0)	1(0.3)
**Sources of Information**				
Mobile	19 (67.9)	30(71.4)	199 (85.4)	248(81.9)
Radio	2 (7.1)	6 (14.3)	20(8.6)	28(9.2)
Newspaper	17 (60.7)	9(21.4)	50(21.5)	76(25.1)
TV without Cable	17 (60.7)	12(28.6)	75(32.2)	104(34.3)
TV with Cable	12 (42.9)	15(35.7)	29(12.5)	56(18.5)

*KIIs and SSIs were not conducted with fathers.

Abbreviations: KIIs = Key Informant Interviews; SSIs = Semi-structured interviews; FGDs = Focused Group Discussions

#### Community Health Workers (n = 67)

The age range of CHWs who participated in KIIs (n = 7) was 24–55 years and had 2–33 years work experience. Sixty CHWs participated in FGDs, 47 from UP and 13 from Bihar. Among these, 19 were ANMs and 41 were ASHAs.

#### Rural Medical Practitioners (n = 8)

The age range of the RMPs was 33 to 65 years with work experience of 3–35 years. Three RMPs were reportedly trained in alternative system of medicine while the rest had no medical qualification. They had learned to give medicines from family members who had no formal medical training themselves.

#### Local illness terms for pneumonia

There were several local terms for serious respiratory illness, including the term “pneumonia”. Pneumonia was used to describe a respiratory illness marked by cough and breathing difficulty. Local terms that encompass signs of clinical pneumonia reported by caregivers from different districts were listed and are available in project website [31]. Semantic analysis revealed that all local illness categories associated difficult breathing more commonly with chest in-drawing than fast breathing.

### Recognition of signs of serious childhood pneumonia

We collected data on both what respondents said were the signs of “pneumonia” as well as danger signs indicating that such a case was serious. We then triangulated this data with what respondents recognized as signs of CAP when shown video vignettes of children having mild, moderate and severe pneumonia.

#### Caregivers (n = 303)

Most caregivers stated that both fast breathing and difficult breathing occurred in cases of “pneumonia”, but some also reported slow breathing. When asked about danger signs of “pneumonia” requiring immediate consultation from a doctor, most respondents mentioned high fever, cold and chest in-drawing. Other IMNCI danger signs such as inability to feed, lethargy, reduced consciousness and convulsions were reported by only few respondents.

During the FGDs, when the respondents were shown the first of the three videos (Table 2), they were unable to recognise fast breathing in the sick child. On the contrary, in most districts, the respondents assumed the child to be healthy and “not suffering from any illness”. In the second video (Table 2), however, most young mothers, some old mothers and few fathers immediately recognised chest in-drawing along with fast breathing. Removing children’s clothing to assess the severity of this illness or to monitor its progression was not a common practice. On the contrary, some women expressed concern about exposing a child’s chest to the wind for fear it would escalate the illness. They kept their children bundled up when ill. “*When fever occurs in winter*, *we wrap the child with warm clothes but when it occurs in summers we wipe the forehead with towel soaked in cold water*. *A strip of cold cloth on the forehead will bring fever down*” (Mother, Gorakhpur (FGD)).

Upon seeing the third video (Table 2), most caregivers stated that the child was “very sick”, but many did not identify the illness as “pneumonia”. Also notable, audible sounds like grunting and groaning were missed by most mothers whereas fathers more often noticed them. In sum, triangulation of data from KIIs, SSIs and the FGDs revealed that: (a) When asked for the signs of pneumonia, fast breathing is reported by most but not all caregivers; (b) Fast breathing is not recognised by many caregivers when presented visually as a standalone sign of CAP. CAP was recognised more commonly when presented with chest in-drawing and difficult breathing; (c) Audible sounds of grunting and groaning were not associated with the severity of CAP presented in illness video vignettes.

#### Community Health Workers (n = 67)

Responses of CHWs on the recognition of signs of pneumonia were almost similar to those given by caregivers. Significantly, many CHWs were also not aware that fast breathing was an early sign of pneumonia. Likewise IMNCI danger signs of reduced consciousness and convulsions were not mentioned by any CHW.

#### Rural Medical Practitioners (n = 8)

Most RMPs reported that “pneumonia” was a common childhood respiratory illness and said they often treated this condition. While some RMPs identified “pneumonia” with difficult breathing, most associated it with fast breathing, unlike the caregivers and community health workers. RMPs were not aware of the IMNCI danger signs.

### Delay in Decision to Seek Health Care

Most mothers felt comfortable consulting local RMP since it was socially acceptable for them to visit the RMP alone. When “pneumonia” was perceived to be more serious, health care decision making became more complicated. Decision to seek care outside the village necessitated consultation with husbands or mothers-in-law or fathers-in-law. Often the grandparents were the primary decision makers. In nuclear families, the disease was identified by the mothers who also took the primary decision to seek care. Mother was usually accompanied by other family member when seeking care for a sick child outside the village. *“Mostly mother and grandfather come with the child*. *You may say it is the rule of the village that the mother will always be accompanied by grandfather*. *Father comes rarely*.*”* (CHW, Mahoba (KII)).

Decision making took into consideration perceptions of severity of illness, availability of transportation, perceived cost of consultations/visits to hospitals and available funds or resources to borrow money. During the FGDs’ respondents pointed out that where primary decision makers were not on site, they were contacted telephonically for advice and directions. This had happened over the last few years since almost all houses processed at least one mobile phone. The mobile phones are usually carried by men, but in emergency women have access to neighbours’ phones also.

### Delay in Seeking Qualified Medical Care

When presented with cases scenarios and videos of different severities of CAP as mentioned in Table 2, the reported care seeking behaviour along with reasons for delay is summarised in Table 4.

**Table 4:** Case management of childhood pneumonia by perceived severity (perceptions of caregivers and care providers).

Illness scenarios/videos (detailed in Table 2)	Respondents	KIIs	SSIs	FGDs	Key Emerging Themes resulting in delay
Illness Scenario 1 & Video 1*	Caregivers	Some in UP would choose RMP or BBD. In Bihar, all would prefer RMP. Few could go to public hospital and few to traditional healer.	Almost half would prefer RMP. Few would wait & watch. Few would go to public hospital. None would go to private hospital.	Most felt that child was not sick hence no treatment required	**Caregivers:** If perceived to be ‘non-sick’, no treatment was required. If perceived to be sick then RMP would be consulted **Care providers:** CHWs as well as RMPs would treat themselves and not refer as child with fast breathing was perceived to be less sick
	CHWs	Most would treat the child themselves. Some would refer to public facility	NA	Most would treat themselves. Some would refer to public hospital.	
	RMPs	Most would prefer to treat the child themselves. Few would send to public facility	NA	NA	
Illness scenario 2 & Video 2*	Caregivers	In UP, few would go to RMPs. Most would use home remedies. In Bihar, most young and all old mothers would go to RMPs. None in U.P. or Bihar would go to private/public hospital.	In UP and Bihar, most would go to BBDs, Few to ANMs, Few to RMPs, Few to Medical college, Few to private hospital, and Few would use home remedy and wait and watch.	Would use home remedy for 1–2 days, then RMPs, then BBD and Medical College. Few to traditional healer as second choice	**Caregivers:** Could use home remedy while they wait & watch. Else could go to RMPs or BBD. **Care providers:** RMPs would treat while the CHWs would refer to public hospital.
	CHWs	Most would refer to public hospital. Few to private hospital	NA	Most would refer to public hospital. Few to higher private or public hospital.	
	RMPs	Almost half would treat the child and would observe for 1–2 days, then refer to public hospital.	NA	NA	
Illness scenario 3 & Video 3*	Caregivers	In UP, most young mothers would go to private hospital while most old mothers would go to public hospital. Some would go to BBD. In Bihar, few young mothers and all old mothers would go to BBD. Few would go to public hospital.	In UP, half would go to private hospital. Some would go to BBD and some to medical college. In Bihar most would go to BBD, some would go to public hospital.	Child was ‘very sick’. Most would go to private hospital or public hospital. Some to BBD.	**Caregivers:** first go to BBD/private hospital and then to a public hospital. **Care Providers:** CHWs would refer directly to public hospital. RMPs would advise to take the child to child specialist or a public hospital
	CHWs	Most would refer to public hospital, few would refer to private doctor	NA	Most would send to public hospital while a few would advise for any specialist (BBD/ private hospital)	
	RMPs	Most would refer to public or private hospital	NA	NA	

*Videos were shown in FGDs only.

NA means that study tool was not used for that category.

Abbreviations: RMPs = Rural Medical Practitioner (usually unqualified). BBD = Block-based doctor (usually qualified). CHWs = Community Health Workers

#### Childhood Pneumonia: Routine Case Management by CHWs

Most CHWs stated that they gave antipyretics to children suffering from pneumonia. However a few also prescribed oral antibiotics (like cotrimoxazole) and anti-tussives. No CHW reported ever prescribing injectable antibiotics, corticosteroids and bronchodilators. CHWs advised the parents to monitor their sick children carefully when receiving treatment. Improvement in child`s condition would be evident when he/she becomes playful, temperature comes down and starts to feed normally. If breathing does not return to normal and there is retraction of ribs it indicates that the treatment was not working. “*If the child is breathing normally*, *is feeding and the temperature is also down*, *then it means there is some improvement in the condition of pneumonia*.*”* (CHW, Darbhanga (KII)).

#### Childhood Pneumonia: Routine Case Management by RMPs

Almost half of the RMPs said that they prescribed oral antibiotics and some reported using injectable antibiotics, anti tussives, corticosteroids and bronchodilators. RMPs who administered injections stated that they did so to keep the “*situation under control*” and because these provided “*fast relief*”. Most RMPs stated that injections were commonly demanded by caregivers. “*Giving injections is important*. *It starts working immediately in the body and controls the disease in 20–30 minutes*. *Although I do not prefer injections*, *but often parents insist that injection should be given*. *Then I have to listen to them*.”(RMP, Darbhanga (KII)). Notably, only some RMPs reported examining sick children they suspected of having “pneumonia” by looking for chest retractions. When RMPs were asked to describe signs of improvement in a child of pneumonia they looked for reduction of fever, cough and breathing rate. “*If the parents are educated*, *the doctor (i*.*e*. *the RMP) tells them to use a thermometer and keep on checking the progress of fever*. *If there is no improvement*, *he calls them again”* (Older caregiver, Meerut, (KII)).

#### Case studies

**Fig 4:**
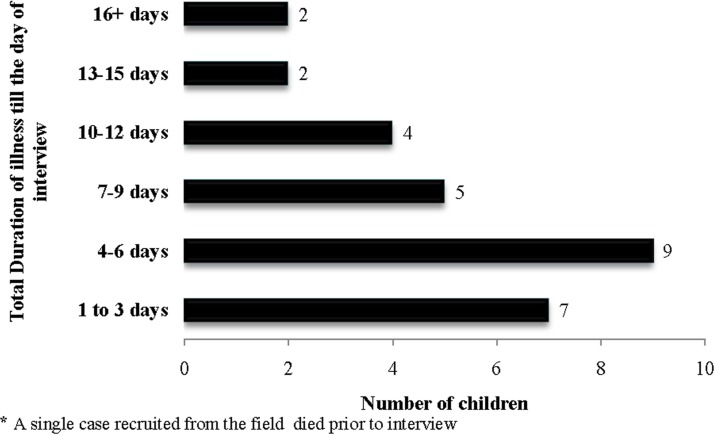
Duration of the illness before reaching the hospital.

An assessment of 30 cases of pneumonia being treated at a tertiary care hospital revealed that in 40% of the cases caregivers had first visited a RMP, 10% a traditional healer, 24% a private qualified block-based doctor (BBD) and 26% a government medical college/hospital/other government facility. Fig 4 depicts the duration of illness before reaching the hospital where the cases were recruited.

#### Private providers and hospitals are frequented more than government hospitals

In this exploratory study, reasons for preferring the RMPs were their round the clock accessibility, familiarity, good treatment outcomes in the past, low charges with an option of credit and acceptance of kind for cash, and a perception of availability of modern essential treatment equipment like thermometers, blood pressure instruments, nebulizers etc. “*Private (block based private doctor) will charge at least Rs*. *300*. *He will again charge Rs*. *300 after 5 days*. *This is just the fees*. *There will be expenses on medicine and investigations as well*” (Father, Meerut (FGD)). Caregivers perceived that private hospitals, though costly, were more effective at times of medical crises. In private facilities, admissions were faster, were less crowded and cleaner and there was round the clock availability of well behaved doctors, nurses and medicines which were lacking in the government sector. Negative perceptions of government medical facilities were related to unavailability/limited availability of necessary medicines and diagnostic tests, the perception that medicines available were of poor quality, overcrowding and referral of critical patients to distant government hospitals. “*The government doctor does not treat us well*. *They do not give medicines*. *Even if they give us*, *and that is rare*, *government medicines do not suit our children”*(Younger Mother, Darbhanga (FGD)).

## Discussion

This study identified several predisposing, enabling and service related factors resulting in delay of appropriate care seeking for CAP. Most prominent factors were lack of recognition of early danger signs of CAP, attempts at self medication using herbal as well as over the counter medications, frequenting RMP first due to a several reasons ranging from easy access by mothers to low charges and easy credit, poorly trained RMPs not referring cases in a timely fashion to hospitals, lapse in decision making due to non availability of husbands or elders, difficulty in arranging transport to clinics in rural areas, time needed to collect funds to pay treatment related expenses, and little trust in government health facilities. An analysis of case studies of CAP revealed that most children had been given medicines at home and half of them had been initially treated by an RMP or a traditional healer. There was a median delay in medical care-seeking of 2 days (range 1–8 days) after caregivers identified illness symptoms as serious.

We found that in UP and Bihar “pneumonia” was a popular illness category connoting serious childhood respiratory illness. However, there was little consensus, about both the signs of “pneumonia” and signs of serious illness. Fast breathing was not universally recognized as an early sign of “pneumonia” and was often recognised only when accompanied by chest in-drawing and difficult breathing. When local terms were used to describe cases of clinical pneumonia, they more often focused on chest in-drawing than fast breathing. Chest in-drawing appeared to eclipse fast breathing as a local indicator of “pneumonia” severity. Similar findings have been reported elsewhere [32–35]. Not appreciating the severity of fast breathing, an early sign of pneumonia, could delay the pursuit of medical care. This was corroborated by our case studies leading us to suggest that newly designed IEC materials should emphasize fast breathing as an early sign of “pneumonia” severity requiring prompt medical care.

Several studies conducted in South Asia have previously reported use of home remedies/self-medication for symptoms of childhood pneumonia [11–12] [36–37]. We also found that home remedies and self medication delayed the pursuit of medical care. Self treatment was often related to scarce resources and concern about the cost of care [38–41]. Also widely reported in the literature on health care seeking in India is the continued popularity of RMP as a first line of resort for illness. Our study of the popularity of RMPs for the treatment of childhood pneumonia corroborates the findings of another recent study carried out in rural UP and Bihar [7]. As observed in other studies [3] [42–44] RMPs are popular despite offering sub-standard care, because they were at once accessible, able to spend more time with their clients compared to practitioners working in the public sector, affordable, and sensitive to the cultural health concerns and medicine preferences of their patients.

Overall, we found that caregivers preferred seeking consultation from village-based RMP for mild to moderate pneumonia, private BBD for severe pneumonia and a town-based private hospital/BBD for very severe pneumonia. We likewise found this to be the case in our study area. It appears that RMPs frequently treat cases of CAP, and have poor knowledge of danger signs which no doubt delays referral of serious cases.

We investigated whether the absence of a primary decision maker could be a factor in health care seeking delay. Mothers of young children generally needed to consult their husbands or mothers-in-law about health care decisions especially if they involved consultations outside their village. Delay due for this reason was recognized as a potential problem, but more common in the past than the present. Notably, recent introduction of and familiarity with mobile phones has made communication easier when a decision maker was not present.

Poor availability of transport has been identified as an enabling factor delaying health care seeking outside one’s village [40–42]. Case studies confirmed this in our study. A recent ambulance program introduced into the rural areas of UP and Bihar has the potential of reducing this transportation problem in the case of a pneumonia related emergency. Free public ambulance services are now available at the block level in each district [45,46]. However, we found that the community was not adequately aware of these services. The community needed to be apprised that these services could be used free of charge for any medical emergency including for children who were very sick.

Community health workers have an important role in CAP outreach education, treatment and triage. We assessed their present knowledge regarding CAP. While ANMs knew some of the signs of pneumonia, most did not appreciate the importance of fast breathing as an early sign of pneumonia and as an indicator of life threatening disease. ANMs seldom gave medicines to patients specific for pneumonia and never administered injectable antibiotics. This is no doubt a major reason they were rarely consulted for childhood pneumonia in our study communities, an observation made by other studies as well [10].The Government of India has released new guidelines for management of childhood pneumonia by the ANMs. These guidelines provide for the treatment with oral amoxicillin or co-trimoxazole for non-severe pneumonia and with first dose of intra-muscular Chloramphenicol and urgent referral to hospital for severe pneumonia. There is a need to train all ANMs as per the new guidelines and ensure the availability of paediatric antibiotics to increase trust in government health care. District-level Household Survey (DLHS-3) reported that that care-seeking from public facilities for childhood ARIs was only 7.4 percent in UP [47]. Implementation of the guidelines could change this poor track record. A recent systematic review suggests that community case management of childhood pneumonia by CHWs was associated with a 32% (0·68, 0·53–0·88) reduction in pneumonia-specific mortality [48].

ASHAs, the frontline health functionaries for basic preventive care, also have an important role to play in CAP identification and referral to ANMs or higher level public facilities. We found that the community did seek information from the ASHAs on childhood illnesses, but ASHAs had limited knowledge about the signs of CAP and its management. The presence of ASHAs in the community could be leveraged such that they could play a valuable role in outreach education on childhood pneumonia. ASHAs interviewed were enthusiastic about learning more about CAP and participated in the piloting of educational messages developed by our team and the subject of a forthcoming publication.

## Limitations

Our research was conducted in rural village settings in five districts of Uttar Pradesh and two in Bihar. The study districts were purposively selected to represent different local dialects. The results of the study are, therefore, generalizable largely to the northern region in India only. Since we recruited cases mostly from tertiary care settings, there could be selection bias. There were more male than female children in our case studies reflecting gender bias in health care seeking in India [49]. In this study we did not present the video of a child without any signs of pneumonia to the FGD respondents. This could be a strength as recognition of illness should not need a comparator for an experienced care provider. On the contrary, this could also be a weakness as those with less experience may have needed a comparator for identification of illness. Since the RMPs were repeatedly being identified as the preferred care provider, on an exploratory basis we interviewed a few of them only. Our findings in this direction are likely to lead to further in-depth qualitative research to understand and possibly optimize the role of RMP in managing CAP in rural settings in north India.

## Conclusions

Each of the factors we have identified as negatively influencing health care seeking for CAP needs to be addressed in future community based childhood pneumonia programs in India. In order for a community-based pneumonia management program to be effective, trust in the public health system needs to be built through health system strengthening and quality improvement. An important finding for future health education programs is that the term pneumonia is used widely enough such that local terms may not be necessary in a next generation of IEC messages. This is significant given the large range of dialects in the region. Given that the term is somewhat of an ambiguous marker for illness associated with difficult breathing in children, there is scope to broaden the public knowledge about signs of pneumonia without encountering confusion resulting from strong pre-existing associations with the term.

There was clearly a need for both educating child caregivers to better recognize the danger signs of CAP and the training of CHWs in community-based management and appropriate triage of childhood pneumonia. Trust in both the diagnostic and treatment skills of health staff and the quality of medications they administer will need to be established. Facilities at the public hospitals need to be more responsive to the needs of the community. Since RMPs are a preferred source of care, strategies to define their role in community wide pneumonia programs needs to be explored further.

## References

[1] Rudan I, Boschi Pinto C, Biloglav Z, Mulholland K, Campbell H. Epidemiology and etiology of childhood pneumonia. Bull World Health Organ.2008; 86: 408–416. 18545744 10.2471/BLT.07.048769 PMC2647437

[2] Walker CL, Rudan I, Liu L, Nair H, Theodoratou E, Bhutta ZA et al Global burden of childhood pneumonia and diarrhoea. Lancet.2013; 381(9875):1405–1416. 10.1016/S0140-6736(13)60222-6 23582727 PMC7159282

[3] Hill Z, Kirkwood B, Edmund K. Family and Community Practices that Promote Child Survival, Growth and Development: A Review of Evidence. World Health Organization, Geneva 2004.

[4] Kallander K, Hildenwall H, Waiswa P, Galiwango E, Peterson S, Pariyo G Delayed care seeking for fatal pneumonia in children aged under five years in Uganda: a case series study. Bull World Health Organ. 2008; 86(5): 332–338. 18545734 10.2471/BLT.07.049353 PMC2647445

[5] Taffa N, Chepngeno G. Determinants of health care seeking for childhood illnesses in Nairobi slums. Trop Med Int Health. 2005;10(3):240–245 15730508 10.1111/j.1365-3156.2004.01381.x

[6] Pillai RK, Williams SV, Glick HA, Polsky D, Berlin JA, Lowe RA. Factors affecting decisions to seek treatment for sick children in Kerala. India Soc Sci Med 2003; 57(5):783–790. 12850106 10.1016/s0277-9536(02)00448-3

[7] May C, Roth K, Panda P. Non-degree allopathic practitioners as first contact points for acute illness episodes: insights from a qualitative study in rural northern India. BMC Health Serv Res 2014; 14:182 10.1186/1472-6963-14-182 24755399 PMC4002199

[8] Darmstadt GL, Syed U, Patel Z, Kabir N. Review of Domiciliary Newborn-care Practices in Bangladesh. J Health Popul Nutr 2006; 24(4):380–393. 17591335 PMC3001142

[9] Madsen HO, Hanehoj M, Das AR, Moses PD, Rose W, Puliyel M et al Costing of severe pneumonia in hospitalized infants and children aged 2–36 months, at a secondary and tertiary level hospital of a not-for-profit organization. Trop Med Int Health 2009; 14(10):1315–1322. 10.1111/j.1365-3156.2009.02374.x 19719464

[10] Geldsetzer P, Williams TC, Kirolos A, Mitchell S, Ratcliffe LA, Kohli-Lynch MK et al The recognition of and care seeking behaviour for childhood illness in developing countries: a systematic review. PLoS One 2014; 9(4): e93427 10.1371/journal.pone.0093427 24718483 PMC3981715

[11] Awasthi S, Verma T, Agarwal M. Danger signs of neonatal illnesses: perceptions of caregivers and health workers in northern India. Bull World Health Organ 2006; 84(10): 819–826. 17128362 10.2471/blt.05.029207 PMC2627499

[12] Awasthi S, Srivastava NM, Pant S. Symptom-specific care-seeking behavior for sick neonates among urban poor in Lucknow, Northern India. J Perinatol.2008; 28 Suppl 2:S69–75. 10.1038/jp.2008.169 19057571

[13] Srivastava NM, Awasthi S, Agarwal GG. Care-seeking behavior and out-of-pocket expenditure for sick newborns among urban poor in Lucknow, northern India: a prospective follow-up study. BMC Health Serv Res.2009; 9:61 10.1186/1472-6963-9-61 19341473 PMC2676263

[14] Walley J, Lawn JE, Tinker A, de Francisco A, Chopra M, Rudan I et al Primary health care: making Alma-Ata a reality. Lancet 2008; 372: 1001–1007. 10.1016/S0140-6736(08)61409-9 18790322

[15] Bhutta ZA, Das JK, Walker N, Rizvi A, Campbell H, Rudan I et al Interventions to address deaths from childhood pneumonia and diarrhea equitably: what works and at what cost? Lancet.2013; 381: 1417–1429. 10.1016/S0140-6736(13)60648-0 23582723

[16] Registrar General of India (2012–13). Annual Health Survey: Uttar Pradesh. First Updation Survey.

[17] Registrar General of India (2011–12). Sample Registration System (SRS) 2012.

[18] Gove S, Pelto GH. Focused Ethnographic studies in the WHO Programme for the control of Acute Respiratory Infections. Med Anthropol 1994; 15(4): 409–424. 8041238 10.1080/01459740.1994.9966102

[19] Nichter M, Nichter M. Acute Respiratory Illness: Popular Health Culture and Mothers Knowledge in the Philippines. Med Anthropol. 1996; 15(4):353–374. 10.1080/01459740.1994.9966099 8041235

[20] Bhattacharyya K. Key Informants, Pile Sorts or Surveys? Comparing behavioural research methods for the study of acute respiratory infections in West Bengal In Anthropology of Infectious Disease: International Health Perspectives. Inhorn MC and Brown PJ. Gordon and Breach Publishers 1997; 211–238.

[21] Thurmond V. The point of triangulation. J Nurs Scholarsh. 2001; 33(3), 254–256 10.1111/j.1547-5069.2001.00253.x 11552552

[22] Igun UA. Stages in Health-Seeking: A Descriptive Model. Soc Sci Med.1979; 13A:445–456. 472772

[23] Kroeger A. Anthropological and socio-medical health care research in developing countries. Soc Sci Med. 1983; 17: 147–161. 6836349 10.1016/0277-9536(83)90248-4

[24] Awasthi S, INCLEN Childnet Zinc Effectiveness for Diarrhea (IC-ZED) Group. Zinc supplementation in acute diarrhea is acceptable, does not interfere with oral rehydration, and reduces the use of other medications: a randomized trial in five countries. J Pediatr Gastroenterol Nutr. 2006; 42(3): 300–305 16540790 10.1097/01.mpg.0000189340.00516.30

[25] World Health Organization. Department of Child and Adolescent Health and Development (CAH). Integrated Management of Childhood Illness. 2008.

[26] Guest G, Bunce A, Johnson L. How Many Interviews Are Enough? An Experiment with Data Saturation and Variability. Field Methods. 2006; 18(1): 59–82.

[27] Thaddeus S, Maine D. Too far to walk: maternal mortality in context **.** Soc Sci Med. 1994; 38(8):1091–1110. 8042057 10.1016/0277-9536(94)90226-7

[28] Miles MB, Huberman AM. Qualitative Data Analysis: an Expanded Sourcebook. 2nd edition Thousand Oaks, CA: Sage Pub 1994.

[29] Ryan GW, Bernard HR. Techniques to identify themes. Field Methods. 2003; 15: 85–109.

[30] Hsieh HF, Shannon SE. Three approaches to qualitative content analysis. Qual Health Res 2005;15(9):1277–1288. 16204405 10.1177/1049732305276687

[31] Fight Pneumonia Website. Available: http://www.fightpneumonia.org/.2014. Assessed 28 December, 2014.

[32] Hussain R, Lobo MA, Inam B, Khan A, Qureshi AF, Marsh D. Pneumonia perceptions and management: an ethnographic study in urban squatter settlements of Karachi, Pakistan. Soc Sci Med. 1997; 45: 991–1004. 9257392 10.1016/s0277-9536(97)00012-9

[33] Irimu G, Nduati RW, Wafula E, Lenja J. Community understanding of pneumonia in Kenya. Afr Health Sci. 2008; 8(2):103–107. 19357759 PMC2584330

[34] Ukwaja KN, Talabi AA, Aina OB. Pre-hospital care seeking behaviour for childhood acute respiratory infections in south-western Nigeria. Int Health. 2012; 4(4):289–294. 10.1016/j.inhe.2012.09.001 24029675

[35] Hildenwall H, Rutebemberwa E, Nsabagasani X, Pariyo G, Tomson G, Peterson S. Local illness concepts—implications for management of childhood pneumonia in eastern Uganda. Acta Trop. 2007; 101(3):217–224. 17374351 10.1016/j.actatropica.2007.02.003

[36] Winch PJ Final report: formative research on newborn care practices in the home and care-seeking for sick newborns in Upazila Mirzapur, Tangail district, Bangladesh PROJAHNMO-II, Upazila Mirzapur, Bangladesh. 2003.

[37] Sreeramareddy CT, Shankar RP, Sreekumaran BV, Subba SH, Joshi HS, Ramachandran U. Care-seeking behaviour for childhood illness- a questionnaire survey in western Nepal. BMC Int Health Hum Rights. 2006; 6: 7 16719911 10.1186/1472-698X-6-7 PMC1543657

[38] Kumar V, Mohanty S, Kumar A, Misra RP, Santosham M, Awasthi S et al Effect of community-based behavior change management on neonatal mortality in Shivgarh, Uttar Pradesh, India: a cluster-randomized controlled trial. Lancet. 2008; 372: 1151–1162. 10.1016/S0140-6736(08)61483-X 18926277

[39] Mohan P, Iyengar SD, Agarwal K, Martines JC, Sen K. Care-seeking practices in rural Rajasthan: barriers and facilitating factors. J Perinatol. 2008;28 Suppl 2: S29–35. 10.1038/jp.2008.167 19057566

[40] Mesko N, Osrin D, Tamang S, Shrestha BP, Manandhar DS, Manandhar M et al Care for perinatal illness in rural Nepal: a descriptive study with cross-sectional and qualitative components. BMC Int Health Hum Rights 2003; 3: 3 12932300 10.1186/1472-698X-3-3 PMC194728

[41] Winch PJ, Alam MA, Akhter A, Afroz D, Ali NA, Ellis AA et al Local understandings of vulnerability and protection during the neonatal period in Sylhet district, Bangladesh: a qualitative study. Lancet. 2005; 366: 478–85. 16084256 10.1016/S0140-6736(05)66836-5

[42] Dongre AR, Deshmukh PR, Garg BS. Perceptions and health care seeking about newborn danger signs among mothers in rural Wardha. Indian J Pediatr. 2008; 75 (4): 325–329. 10.1007/s12098-008-0032-7 18536884

[43] Kelly L, Black R. Research to support household and community IMCI. Report of a meeting, 22–24 January 2001, Baltimore, Maryland, USA. J Health Popul Nutr. 2001; 19: S111–1148. 11503353

[44] Bojalil R, Kirkwood BR, Bobak M, Guiscafre H. The relative contribution of case management and inadequate care-seeking behaviour to childhood deaths from diarrhoea and acute respiratory infections in Hidalgo, Mexico. Trop Med Int Health. 2007; 12 (12): 1545–1552. 18076563 10.1111/j.1365-3156.2007.01963.x

[45] National Health Mission. Government of Uttar Pradesh. 108 Samajwadi Swasthya Sewa in Uttar Pradesh. Available at http://upnrhm.gov.in/emts108.php

[46] National Health Mission. Government of Uttar Pradesh. 102 National Ambulance Service in Uttar Pradesh. Available at http://upnrhm.gov.in/emts108.php

[47] District Level Household Survey-3: 2007–08. Uttar Pradesh.

[48] Das JK, Lassi ZS, Salam RA, Bhutta ZA. Effect of community based interventions on childhood diarrhea and pneumonia: uptake of treatment modalities and impact on mortality. BMC Pub Health. 2013; 13 Suppl 3: S29. 24564451 10.1186/1471-2458-13-S3-S29 PMC3953053

[49] Mohan Srivastava N, Awasthi S, Mishra R. Neonatal Morbidity and care-seeking behavior in urban Lucknow. Indian Pediatr. 2008; 45(3): 229–23 18367771

